# Alkali fusion using sodium carbonate for extraction of vanadium and tungsten for the preparation of synthetic sodium titanate from spent SCR catalyst

**DOI:** 10.1038/s41598-019-48767-0

**Published:** 2019-08-23

**Authors:** In-Hyeok Choi, Gyeonghye Moon, Jin-Young Lee, Rajesh Kumar Jyothi

**Affiliations:** 1 Convergence Research Center for Development of Mineral Resources (DMR), Korea Institute of Geoscience and Mineral Resources (KIGAM), Daejeon, 34132 South Korea; 2 R&D Center, HANNAE FOR T (Metal Recycling Company), 22, Sandan 3-ro 5-gil, Seongmun-myeon, Dangjin-si, Chungcheongnam-do 31702 South Korea

**Keywords:** Pollution remediation, Energy science and technology, Chemical engineering

## Abstract

Alkali fusion method to extract vanadium and tungsten from spent SCR catalyst and to simultaneously prepare synthetic sodium titanate for the purpose of preparation of feedstock for TiO_2_ manufacturing by hydrometallurgical processing was investigated. Based on the Na_2_O-TiO_2_ phase diagram and experimentally obtained results, appropriate alkali-fusion temperature and molar ratio (MO_x_/Na_2_O + MO_x_) were determined to prepare soluble vanadium, tungsten salts, and sodium titanate. As results, the extraction efficiency of vanadium and tungsten was >99% and sodium titanate as feedstock for making TiO_2_ was obtained under the following alkali fusion conditions: Temperature, 950 °C; reaction time, 20 min; molar ratio, 0.5. When the molar ratio (MO_x_/Na_2_O + MO_x_) was less than 0.5, complete liquidized product (eutectic melt of the feedstock and Na_2_CO_3_) was obtained at the given conditions. This method, compared to conventional alkali roasting, is advantageous for rapid synthesis of water-soluble compounds for leaching of vanadium and tungsten, and for use as feedstock for the preparation of TiO_2_ by hydrometallurgical route.

## Introduction

Since the global demand for V_2_O_5_-WO_3_/TiO_2_ (herein SCR) catalysts has been increasing due to the interest in nitrogen oxide (NO_x_) control, spent SCR catalysts have been contributing to the generation of a significant amount of solid waste^1^^,R1^. In particular, the demand for SCR catalysts in stationary applications, such as thermoelectric power plants, incinerators, and marine systems is known to be significant^2^^,R2^. SCR catalysts for stationary applications usually consist of 70–80 mass% of TiO_2_ (anatase) as a supporting oxide. Vanadium as a catalytic agent and various promoters such as W, Si, and Ca are also used^3^^,R2^. Despite the main catalytic reaction of vanadium for denitrification in a SCR catalyst, a high content (7–10 mass% as WO_3_,) of tungsten is usually chosen over the content of vanadium (0.5–1.5 mass% as V_2_O_5_) due to an improvement in chemical durability of the catalysts.

Owing to stringent regulations for NO_x_ emissions and increasing global demands of SCR catalysts, studies on the recycling of spent SCR catalysts have reported their high potential as a new secondary source^4–8^. Direct leaching of metal in spent SCR catalysts using acid or alkaline agent has revealed as inadequate for effective extraction of vanadium and tungsten^7^. This seems to be due to the characteristics related to the formation of vanadium and tungsten of SCR catalyst. Despite the high-energy consumption process, alkali roasting process is good economic and technical option for metal reclamation from various feedstock due to its simple operation, high yield, and dissolution of insoluble metals^9^. The alkali roasting process is known to be the most practical process to dissolve valuable metals from various feedstock^10–12^. Choi *et al*.^5^ proposed a soda roasting and water leaching process to dissolve vanadium and tungsten. According to their results, vanadium and tungsten from spent SCR catalyst were dissolved with increasing Na_2_CO_3_ addition amount and roasting temperature. However, due to the formation of insoluble calcium compounds of vanadium and tungsten (calcium vanadate and CaWO_4_) in the roasting process, the dissolution efficiency of vanadium and tungsten in water leaching was insufficient within the investigated conditions. In addition, for the completion of an alkali fusion reaction, it took hours in general. Another disadvantage of the previous research was the wasting of TiO_2_ as a residue. Spent SCR catalyst typically contains a considerable amount (70–80 mass%) of TiO_2_ as a supporting oxide. Considering the complexity of upgrading titanium ore or slag for titanium processing^R3,^^13^, the recycling of TiO_2_ using spent SCR catalysts is worthwhile and attractive for producing pigment-graded TiO_2_. Among the various feedstock for producing TiO_2_, sodium titanate (NaTiO_x_) as a promising feedstock has been reported for preparation of iron-free TiO_2_ by a hydrometallurgical route^14–16^. This newly developed process is known to considerably reduce energy consumption compared to the conventional processes of TiO_2_ manufacturing.

This paper addresses the synthesis by alkali fusion of vanadium, tungsten, and titanium with their eutectic melt of spent SCR catalyst and Na_2_CO_3_. For the homogeneity and rapid synthesis of vanadium, tungsten, and titanium, the present work mainly focuses on the optimum synthesis temperature and the Na_2_CO_3_ addition amount for the eutectic system of Na_2_O-MO_x_ (M = Ti, Si, and W in the feedstock). From our study, it is expected that energy saving and more highly efficient alkali fusion process for high yield of vanadium and tungsten is possible. In addition, the preparation of sodium titanate as feedstock for the hydrometallurgical production of TiO_2_ is developed simultaneously.

## Results and Discussion

### TG and DTA results of the feedstock, Na_2_CO_3_ and their mixed samples

The faded lines in Fig. 2 represent the TG-DTA results of the feedstock (solid line) and Na_2_CO_3_ (dashed line) as background. For the TGA and DTA results of Na_2_CO_3_ in Fig. 2(a,b), the typical thermal behavior of pure Na_2_CO_3_ is shown^17,18^. For the feedstock in the TG analysis, about 8% of weight loss was appeared throughout the temperature range, and this result might be explained by the thermal decomposition of unknown matters in the feedstock and it is open for discussion.

**Figure 1:**
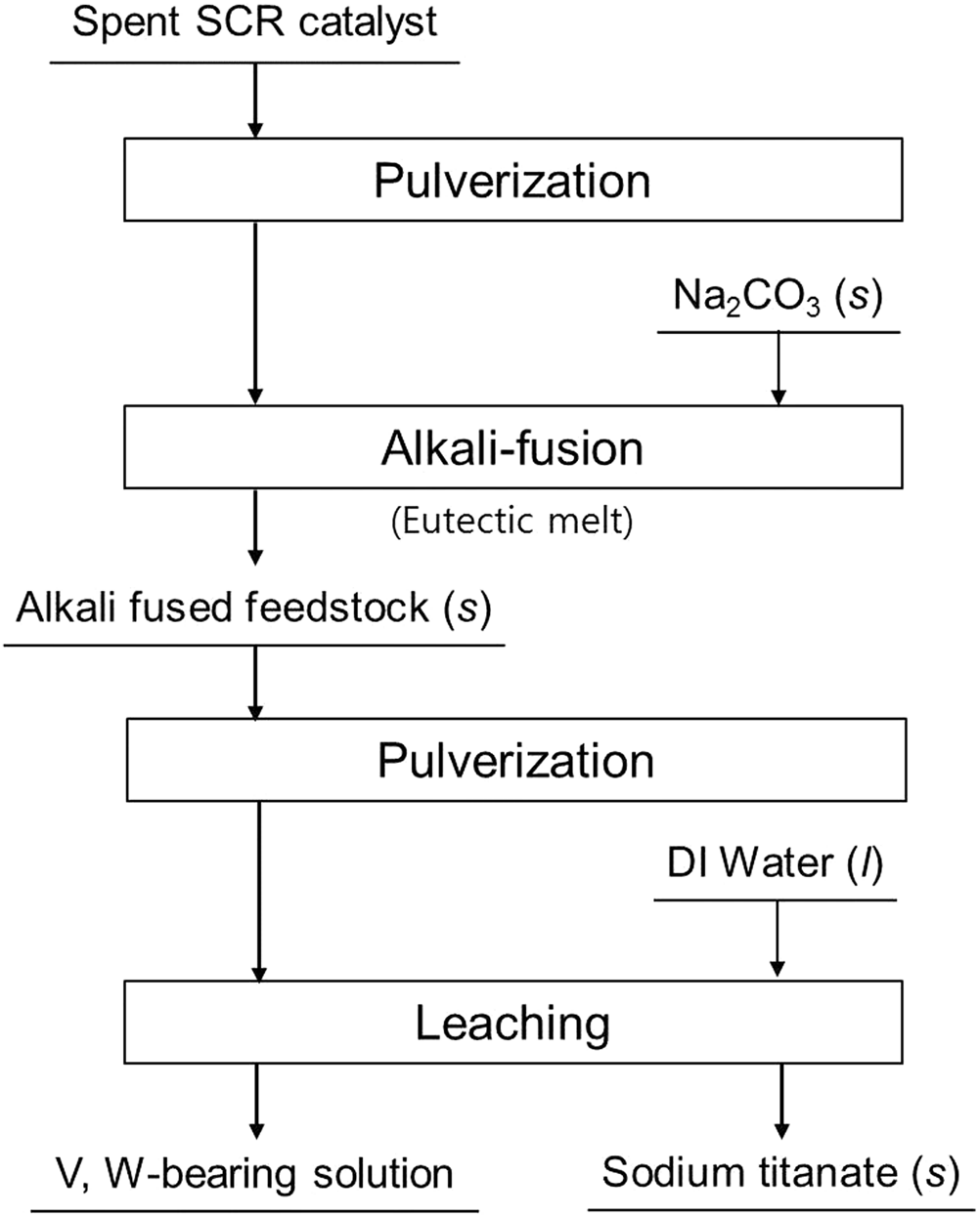
Schematic process flow chart of this study.

**Figure 2:**
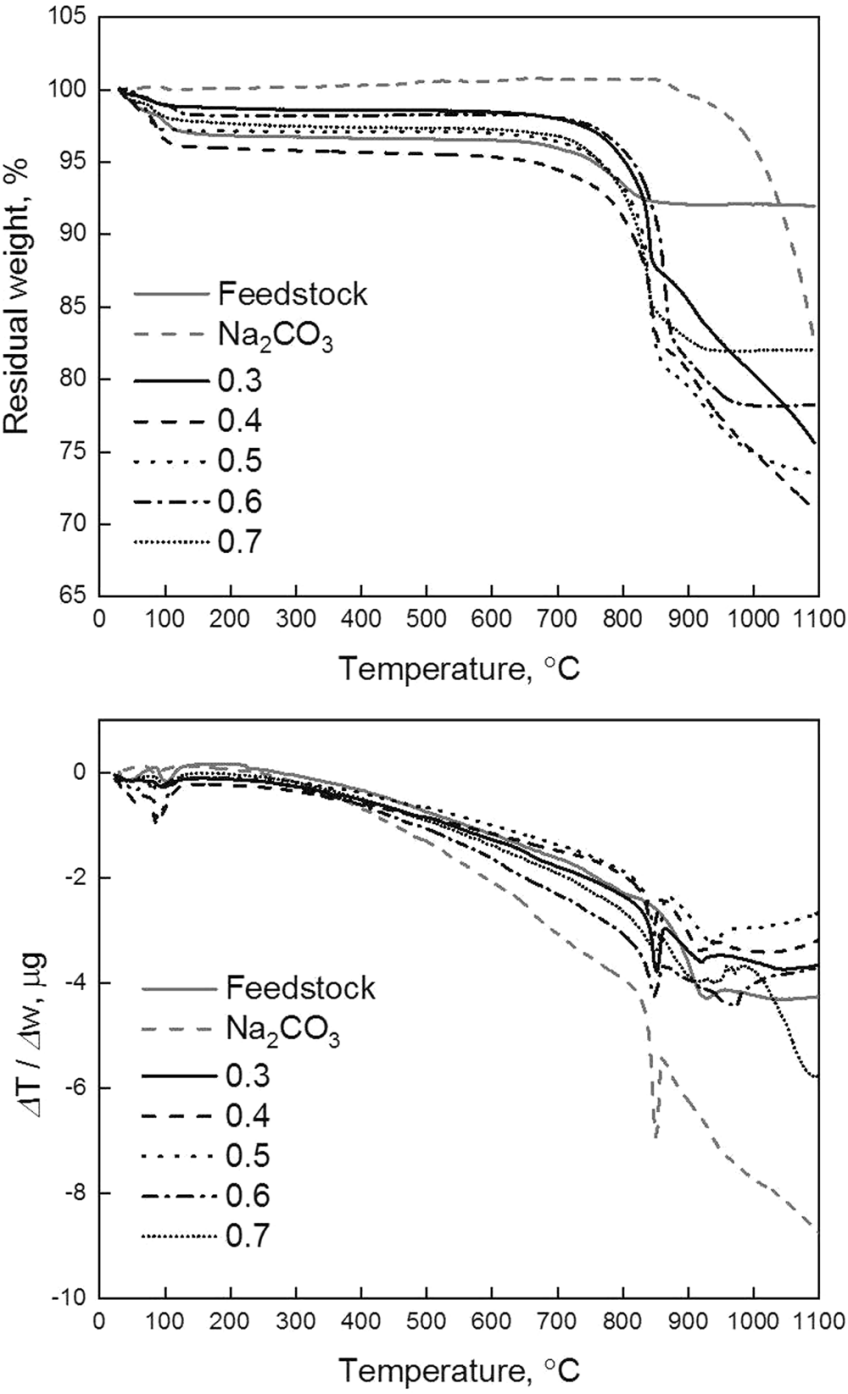
TGA, (**a**), and DTA, (**b**), curves for various mole fraction (MO_x_/MO_x_ + Na_2_O, M = Ti, Si, and W) of feedstock and Na_2_CO_3_.

**Figure 3:**
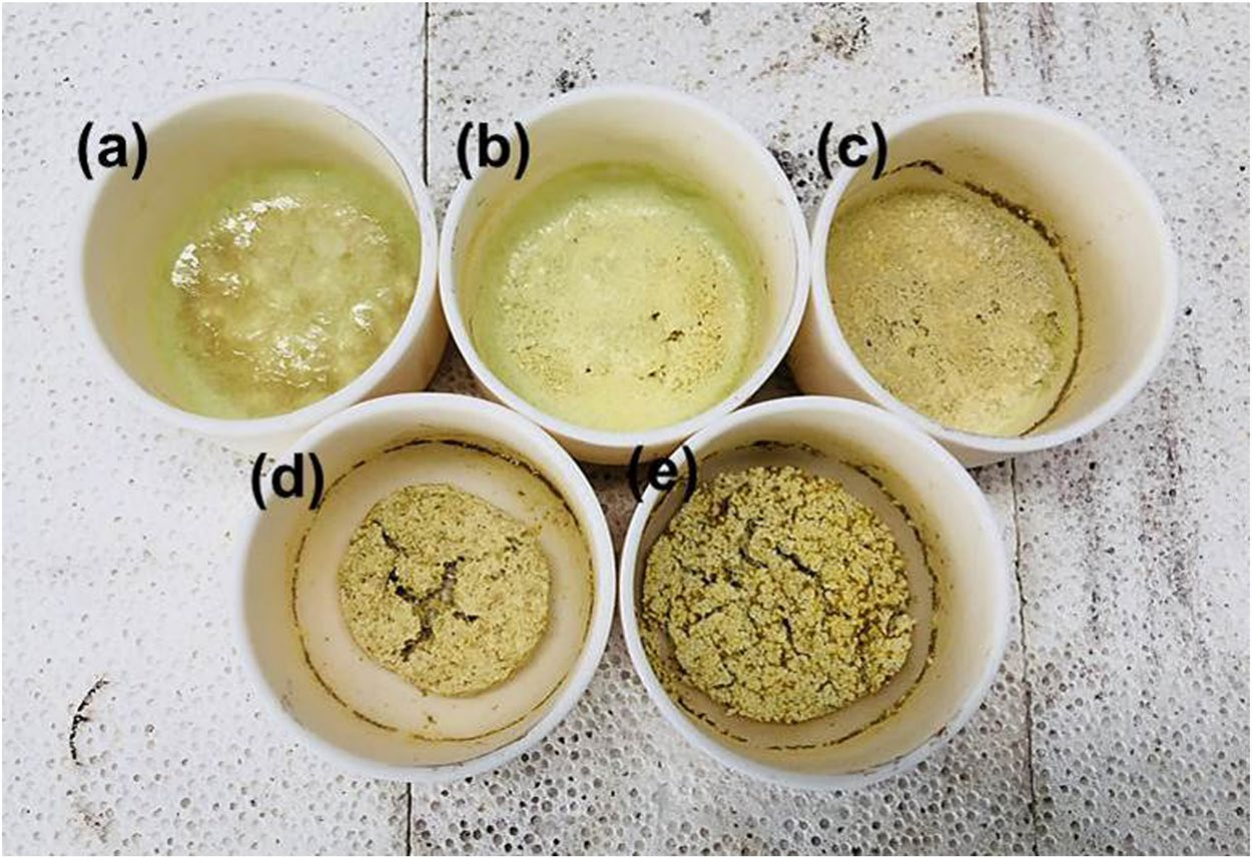
Actual appearances of samples after the alkali-fusion reaction depending on a molar ratio (MO_x_/Na_2_O + MO_x_, M = Ti, Si, W in the feedstock); (**a**) 0.3, (**b**) 0.4, (**c**) 0.5, (**d**) 0.6, (**e**) 0.7 for 20 min at 950 °C.

**Figure 4:**
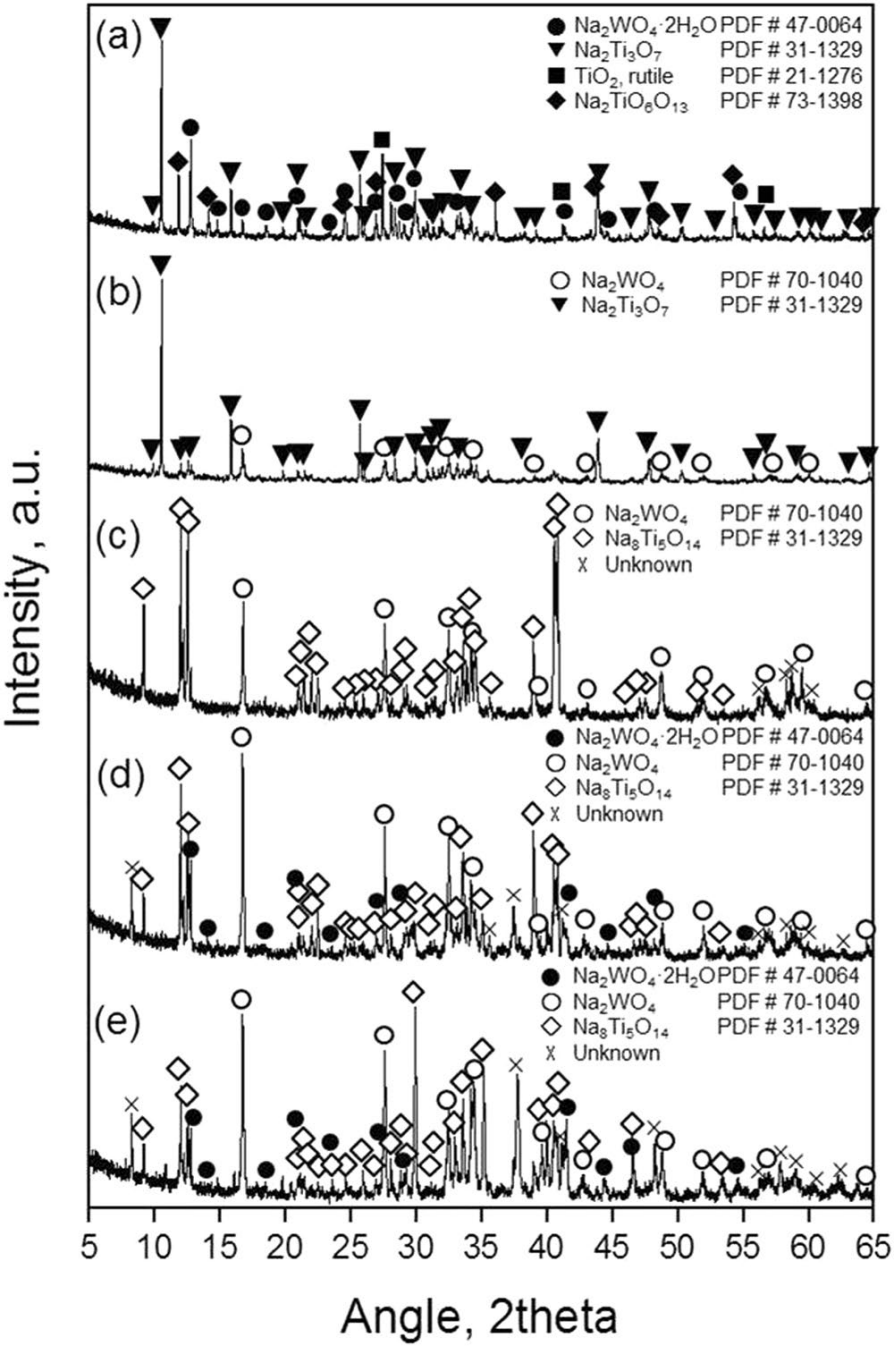
XRD patterns of alkali-fused samples depending on molar ratio (MO_x_/Na_2_O + MO_x_); (**a**) 0.7, (**b**) 0.6, (**c**) 0.5, (**d**) 0.4, (**e**) 0.3.

**Figure 5:**
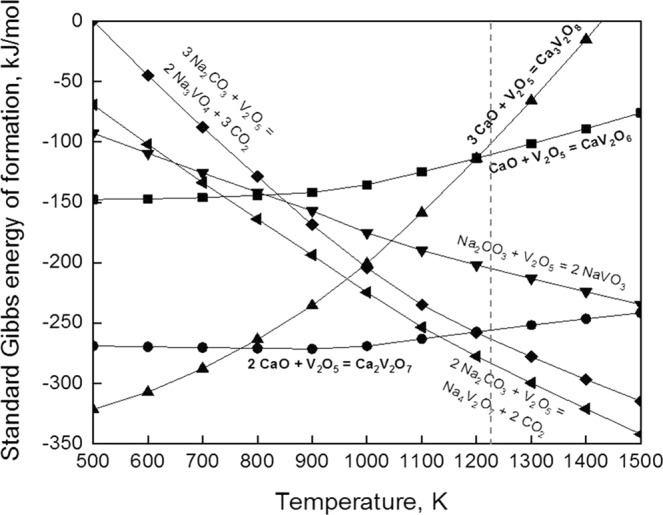
Standard Gibbs energy of formation for some selected vanadium compounds.

**Figure 6:**
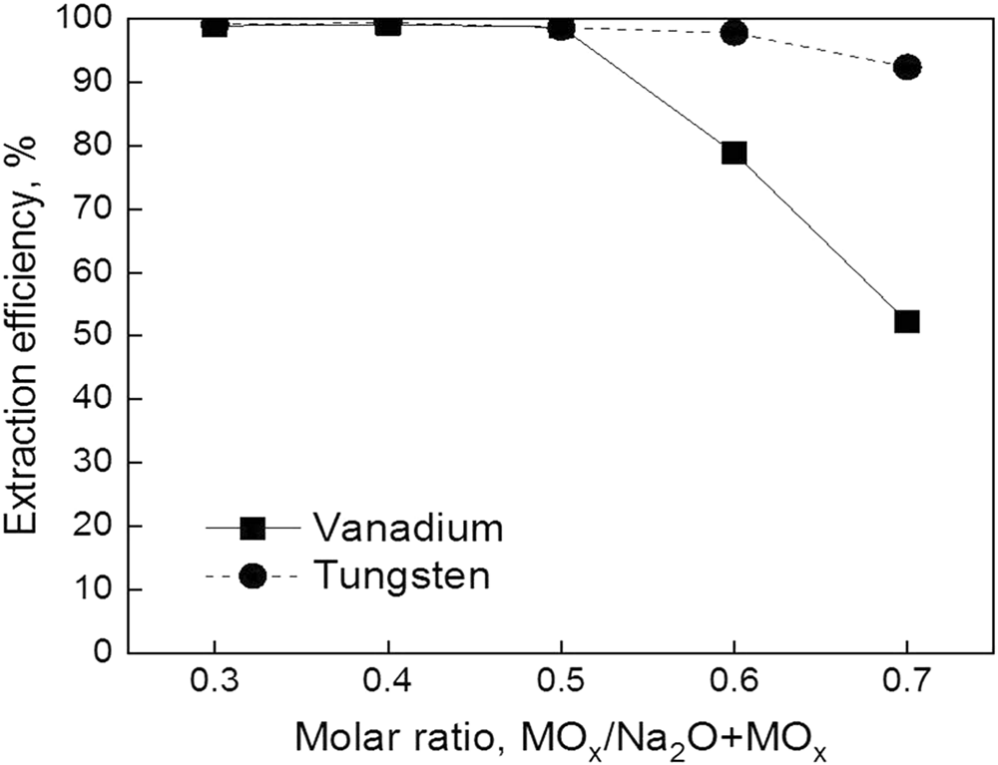
Extraction efficiency of vanadium and tungsten depending on molar ratio in an alkali fusion reaction (Leaching condition: solvent, water; particle size distribution, > 106 μm; temperature, ambient temperature; S/L(W/V), 0.1; time, 2 h).

A noticeable result in the TG and DTA analysis of the feedstock is the endothermic peak in accordance with a decrease of residual weight in the range between 700 and 850 °C. According to a previous study^5^, transformations were initiated with metals including tungsten and titanium into equilibrium form and rutile, respectively, in that temperature range. This endothermic heat flow could be assumed to be enthalpy relaxation of amorphous materials (VO_x_ and WO_x_ on TiO_2_ surface) due to the increase of density and decrease of free volume^19^. On the other hand, decrease of residual weight at 700–900 °C was found. Considering the structure of vanadium and tungsten on the TiO_2_ surface in a SCR catalyst and behavior of phase transformation of supporting oxide (TiO_2_, anatase)^3^, it assumed that decomposition of hydroxyl radicals, which is absorbed in manufacturing of SCR catalysts on the surface of TiO_2_, occur in accordance with phase transformation of anatase into rutile^20^.

Since various metal oxides existed in the feedstock, establishment of eutectic system in this study is challenging. However, based on the Na_2_O-TiO_2_ eutectic system, the eutectic melting point can be roughly derived because the main composition is TiO_2_ in the feedstock. According to the Na_2_O-TiO_2_ phase diagram^21^, various sodium titanate compounds can form including Na_2_TiO_3_, Na_4_TiO_4_, Na_8_Ti_5_O_14_, and Na_2_Ti_3_O_7_, depending on the molar ratio (TiO_2_/Na_2_O + TiO_2_). In addition, the eutectic melting point for the Na_2_O-TiO_2_ system can be decreased with the decrease of molar ratio in the investigated experimental conditions (molar ratio, 0.3–0.7). On the other hand, considering the Na_2_O-SiO_2_^22^^,^ the eutectic melting point using spent SCR catalyst and the Na_2_CO_3_ system can be affected. In particular, based on the Na_2_O-SiO_2_ binary phase diagram, eutectic melts of two phases is relatively high compared to titanium or tungsten at an identical molar ratio. Since silicon in the feedstock is another main component apart from titanium, the eutectic melting point of the feedstock and the Na_2_CO_3_ can be increased.

The TGA results for various samples depending on molar ratio showed similar decreasing tendencies of weight, and two noticeable decreases of weight loss were found. One is evaporation of volatile matter (possibly appearing with residual water) in the feedstock at the initial stage. Another is the remarkable decrease at around the melting point of Na_2_CO_3_. In general, the decomposition of pure Na_2_CO_3_ at its melting point is not significant^17,18^. In order to increase the decomposition rate of Na_2_CO_3_, the temperature should be increased far beyond the melting point of Na_2_CO_3_^17,18^. Thus, when the temperature increases, possible reactions in alkali fusion between Na_2_CO_3_ and MO_x_ can be expressed as follows:

$${{\rm{Na}}}_{2}{{\rm{CO}}}_{3}(s)={{\rm{Na}}}_{2}{\rm{O}}\,(s,l)+{{\rm{CO}}}_{2}(g),\,{{\rm{Na}}}_{2}{\rm{O}}\,(s,l)+{{\rm{MO}}}_{{\rm{x}}}(s)={{\rm{Na}}}_{2}{{\rm{MO}}}_{{\rm{x}}+1}(s,l)$$

$${{\rm{MO}}}_{{\rm{x}}}(s)+{{\rm{Na}}}_{2}{{\rm{CO}}}_{3}(l)={{\rm{Na}}}_{2}{{\rm{MO}}}_{{\rm{x}}+1}(s,l)+{{\rm{CO}}}_{2}(g)$$

where M indicates metals (Ti, Si, W, V, etc.) in the feedstock.

Based on the TGA results in Fig. 2, the decrease of the residual weight of the samples is negligible (<1%) up to the melting point of Na_2_CO_3_. This means that alkali fusion reaction rarely proceeded, or slow below the melting point of Na_2_CO_3_. However, from the melting point of Na_2_CO_3_, the residual weight of the samples significantly decreased with sharp endothermic peaks (Fig. 2(b)) for all conditions. Thus, the decomposition of Na_2_CO_3_ was promoted by the alkali fusion reaction with MO_x_ (M = Ti, Si, W, etc.) in the feedstock and the equilibrium reaction in Eq. 2 is considered to be the main reaction in the alkali fusion process. In addition, alkali-fusion reaction showed significantly fast kinetics when the reaction underwent with solid-liquid reaction.

On the other hand, when the molar ratio was 0.3 and 0.4, Na_2_CO_3_ the decrease of residual weight appeared to be saturated. This means that the alkali-fusion reaction was completed. However, when molar ratio is more than 0.5, alkali fusion reaction was in process from the TGA results with continuous exothermic heat flow. Based on the TG-DTA results in Fig. 2 and the phase diagram of Na_2_O-TiO_2_, the alkali fusion temperature for attaining complete melt was considered to be above 950 °C.

Figure 3 shows the actual appearance of samples after alkali fusion reaction at 950 °C for 20 min depending on the molar ratio of Na_2_CO_3_ and the feedstock. According to the images, sample (a), (b), and (c) are found to be completely melted and liquidized by the given conditions and the molar ratio of Na_2_CO_3_ and the feedstock. Sample (d) was partially liquidized with remarkable volume reduction. However, sample (e) showed a simply agglomerated form without noticeable changes, and was easy to crush and grind. In summary, based on the observations, in order to make eutectic melts with the feedstock and Na_2_CO_3_ for fast synthesis, 0.5 molar ratio appeared to be most suitable for the given alkali fusion conditions.

### XRD patterns and their variation of fused samples

Figure 4 shows the XRD patterns for the alkali-fused samples. A noticeable result is that the non-melted sample, (a), shows the various sodium titanate compounds in accordance with unreacted TiO_2_. Based on the dissolution efficiency results, the XRD patterns of the partially melted sample, (b), appear to show that titanium and tungsten were completely alkali-fused. The fully melted samples, (a), (b), and (c), were shown with Na_8_Ti_5_O_14_ phase, and this phase can be appeared when a molar ratio from 0.525 to 0.725 is based on the Na_2_O-TiO_2_ binary phase diagram. This chemical formula was maintained despite the increase of Na_2_CO_3_ amount. Moreover, even when the molar ratio was 0.3, the chemical formation of sodium titanate remained unchanged. It seemed that Na_2_TiO_3_ was polymerized into Na_8_Ti_5_O_14_ and Na_2_O^23^. However, this metastable phase of sodium titanate can be utilized for follow-up process (acid leaching).

At the low molar ratio (0.6 and 0.7), sodium metatitanate (Na_2_Ti_3_O_7_) appeared. Although Na_2_Ti_3_O_7_ is soluble in acid solution, it is even when finely ground, and it only very slowly dissolves^24^. Thus, Na_2_Ti_3_O_7_ is not adequate for feedstock for the preparation of TiO_2_ by hydrometallurgical route. Further, when the molar ratio was 0.7, unreacted TiO_2_ was found. Considering the hydrometallurgical processing including acid leaching and hydrolysis of the feedstock, the molar ratio of the feedstock and Na_2_CO_3_ should be higher ratio of Na_2_CO_3_ ( < 0.5) for the appropriate synthesis of TiO_2_ and liquidation (eutectic melt).

Tungsten in the feedstock was characterized by Na_2_WO_4_ (and its hydrated form) by XRD analysis. It can be observed that the peak intensity of Na_2_WO_4_ was developed in accordance with a decrease of molar ratio. This compound easily dissolves in water with high solubility. Silicon, one of the main components in the feedstock, was not observed including its alkali-fused compounds. In addition, vanadium, one of the target materials, was not present.

Based on the XRD results of each of the alkali-fused samples, when the molar ratio of the feedstock and Na_2_CO_3_ was 0.5 or less, water-soluble Na_2_WO_4_, in accordance with acid-soluble sodium titanate, could instantly be obtained by making their eutectic melts. Considering the conventional process (alkali roasting), homogeneous synthetic products can be obtained with less reaction time. This also leads to energy-saving alkali-fusion process.

### Leaching efficiency of vanadium and tungsten in alkali-fused samples

In the previous report^7^, one reason for the low leaching efficiency of vanadium and tungsten in alkali-roasted feedstock by water leaching is the formation of insoluble compounds including calcium tungstate and calcium vanadates in the alkali roasting of spent SCR catalyst. Detrimental effect of insoluble formation for extracting vanadium was significant. These formations originated from the heterogeneous alkali roasting reactions (side reaction including insoluble calcium-vanadium compounds) and low activity of Na_2_CO_3_. In particular, the leaching efficiency of vanadium was remarkably low compared to tungsten because of high driving force of the calcium compound formations (Fig. 5). In addition, it appeared that a degree of calcium vanadate formation in alkali roasting was determined by the amount of CaO in the feedstock regardless of the Na_2_CO_3_ addition amount according to a previous report^5^. However, based on the Na_2_O-CaO-V_2_O_5_ ternary phase diagram^25^, soluble sodium vanadate species are possibly formed due to sufficient Na_2_CO_2_ (Na_2_O) activity and negligible CaO content in the mixture.

In contrast, the activity of Na_2_CO_3_ in the mixture is relatively sufficient in our investigation. Also, when the molar ratio was 0.5 or less, the residual weight in the TGA results was continuous. Thus, the activity of CO_3_^2−^ in the leaching solution assumed to be high. When the [CO_3_^2−^] existed in aqueous solution (alkaline area, pH < 14), calcium vanadate can be dissolved as below equilibrium reaction:

$${{\rm{CaV}}}_{2}{{\rm{O}}}_{6}(s)+{{\rm{Na}}}_{2}{{\rm{CO}}}_{3}(aq)=2{{\rm{NaVO}}}_{3}(aq)+{{\rm{CaCO}}}_{3}(s)$$

Based on the TG-DTA results, when the molar ratio was below 0.5, the decrease of residual weight was not completed. This means that free Na_2_CO_3_ existed in the alkali-fused sample. Thus, in the water leaching of the alkali-fused samples, existence of [CO_3_^2−^] is possible in the leaching solution. This condition is favorable for a dissolution of calcium vanadate.

On the other hand, when the molar ratio was 0.5 or below, the pH of the solution after water leaching of the alkali-fused samples appeared to be highly alkaline. According to the measured pH value (calibration between pH 4.01 and 12.46, *R* = 99.998), high alkalinity may have originated from the following equilibrium reaction in the water leaching:

$${{\rm{Na}}}_{8}{{\rm{Ti}}}_{5}{{\rm{O}}}_{14}(s)+{\rm{9}}\,{{\rm{H}}}_{2}{\rm{O}}\,(l)=5\,{{\rm{H}}}_{2}{{\rm{TiO}}}_{3}(s)+{\rm{8}}\,{\rm{NaOH}}\,(aq)$$

This leaching environment is also favorable for dissolving insoluble calcium vanadate. Thus, even if low activity of CO_3_^2−^ is in the solution, the leaching condition for the dissolution of calcium vanadate compounds is fulfilled due to the high alkalinity (pH > 14). However, the specified leaching mechanism and equilibrium formation of vanadium in alkali fusion process is open for discussion.

Figure 6 shows the results of leaching efficiency of each alkali-fused sample in water. Based on the results, the leaching efficiency of vanadium and tungsten was saturated to >99% at ≥0.5 molar ratio or less. Dissolved titanium in water leaching was below 2 mg·L^−1^ for all conditions. Compared to previously reported alkali roasting and other conventional processes, the alkali fusion method attained remarkably high extraction efficiency of vanadium and tungsten with shortened reaction time. The influence of the molar ratio on the leaching efficiency of vanadium was remarkable. With less Na_2_CO_3_ addition (molar ratio = 0.6, 0.7), a significant decrease of leaching efficiency of vanadium appeared. Based on the solubility of calcium vanadate in high-alkaline solution (pH > 14) and the measured pH of the water leaching solution, the dissolution of calcium vanadate appeared to relate to the the pH of leaching solution (high alkalinity).

## Experimental

### Sample preparation and chemicals

The feedstock (spent SCR catalyst) used in this study was supplied by Samcheonpo thermoelectric power plant, South Korea. Attached dust, ash, and poisons in the feedstock were firstly removed. Then the feedstock was pulverized by jaw crushing and mill grinding. The particle size distribution of the pulverized feedstock was determined (mainly ranging from 1 to 100 μm) by particle size distribution analysis (Mastersizer2000, Malvern Instruments, USA). Pulverized feedstock was dried at 80 °C in an oven over 24 h to remove residual moisture. Apart from the aforementioned process, further pre-treatment was not conducted. Table 1 shows the chemical composition of spent SCR catalyst as feedstock. Na_2_CO_3_ (99.8%, Kanto Chemicals, Japan) was used as an alkali agent for the alkali fusion reactions.

**Table 1:** Chemical analysis of feedstock (Spent SCR catalyst composition).

Composition	TiO_2_	SiO_2_	WO_3_	CaO	Al_2_O_3_	V_2_O_5_	Fe_2_O_3_	MgO	MoO_3_	H_2_O
wt%	70.9	9.32	7.11	2.50	2.41	0.98	0.25	0.13	0.12	4.5

### Experimental conditions and analysis

The mixing ratio varied between 0.3–0.6 (mole fraction ratio = MO_x_/Na_2_O + MO_x_, M = Ti, Si, and W) to determine the eutectic melting point of the mixture (pulverized feedstock and Na_2_CO_3_). The alkali fusion time was fixed at 20 min for all mixed samples. Process flows, which is a sequence with pulverization, alkali fusion, and water leaching, of this study is shown in Fig. 1. Simultaneous thermogravimetric analysis (TGA) and differential thermal analysis (DTA) for the various mixtures were carried out using a DTG-60H (Shimadzu, Japan) analyzer from ambient to 1100 °C in air. The heating rate was 10 °C min^−1^. In order to investigate the leaching efficiency of vanadium, tungsten and titanium in each fused feedstock, water leaching was conducted under the following conditions: Temp., ambient temperature; S/L (V/W) ratio, 0.2; time, 2 h; particle size distribution, ≥70 mesh (<210 μm).

Phase characterization of the fused samples and residues was carried out using an X-ray diffractometer (XRD, X’pert MPD, Phillips, USA). pH measurement was done using Orion Versa Star (Thermo Scientific, USA).

## Conclusions

In order to develop the process of extraction of vanadium and tungsten in accordance with a preparation of synthetic sodium titanate from spent SCR catalyst, the alkali fusion method was investigated in our study and our conclusions are presented here. The alkali fusion method using spent SCR catalyst and Na_2_CO_3_ was newly developed for the extraction of vanadium and tungsten in accordance with the preparation of sodium titanate as feedstock for the production of TiO_2_ by hydrometallurgical route. Based on the TG-DTA results, alkali-fusion conditions for the eutectic melt of spent SCR catalyst and Na_2_CO_3_ were established under the following conditions: Molar ratio, 0.5; temperature 950 °C; time, 20 min. Under these conditions, the extraction efficiency of vanadium and tungsten from spent SCR catalyst was >99% (Ti, >2 mg·L^−1^). In addition, sodium titanate was obtained for the preparation of TiO_2_ by hydrometallurgical processing. This investigated method can be considered as an alternative process for the efficient and energy-saving extraction process of vanadium and tungsten as well as simultaneously for the preparation of sodium titanate as a TiO_2_ source, with shortened reaction time and homogeneous reactions.
